# Impact of connected dispensing technology with advanced analytics in a multicenter health system

**DOI:** 10.1093/ajhp/zxae198

**Published:** 2024-07-16

**Authors:** Steven Freeman-Muhammad, Regina Chipman-Ashley, Richard E Martin, Jennifer Williams, Amanda Prochazka, Doina Dumitru, Craig Greszler

**Affiliations:** Novant Health, Winston-Salem, NC, USA; Novant Health, Winston-Salem, NC, USA; Novant Health, Winston-Salem, NC, USA; Novant Health, Winston-Salem, NC, USA; UNC Health, Chapel Hill, NC, USA; Becton, Dickinson and Company, San Diego, CA, USA; Becton, Dickinson and Company, San Diego, CA, USA

**Keywords:** ADC, automation, carousel, inventory management, procurement, supply chain

## Abstract

**Purpose:**

This study was designed to evaluate the impact of enterprise inventory optimization (EIO) technology and analytics on pharmacy labor, costs, and medication availability within a large integrated delivery network (IDN).

**Methods:**

This article describes a mixed-methods, postmarket observational study assessing the impact of a solution of disparate technologies including automated dispensing cabinets (ADCs), centralized pharmacy inventory software, and controlled substance vaults connected by an inventory optimization analytics (IOA) tool. Four study modules were implemented over a 10-month period. The intervention consisted of implementation of the IOA software, linking the disparate automated technologies. Transactional data was collected and aggregated with user perception survey data in both the pre- and postintervention periods. Descriptive and comparative statistical testing was used to assess outcomes.

**Results:**

A total of 11 facilities with bed counts ranging between 22 and 908 beds were included in this study. At an enterprise level, users were able to complete an average of 2.8 times more periodic automated replenishment (PAR) level changes post intervention, resulting in an estimated enterprise labor avoidance of over 1 full-time equivalent (2,099 labor hours) annually. Despite an enterprise decision to increase ADC inventory on hand from a 3-day supply to a 5-day supply, 5 sites (45%) had a decrease in total inventory, while 9 sites (82%) saw a decrease in ADC inventory costs. Additionally, 7 sites (64%) saw a reduction in the ADC stockout percentage and all 11 sites (100%) saw a decrease in the central pharmacy stockout percentage post intervention.

**Conclusion:**

Integration and optimization of connected inventory management technology was observed to have positive impacts on improving labor productivity, reducing ADC inventory carrying costs, and increasing medication availability.

Recently, healthcare systems have experienced financial pressures reshaping how patient care is delivered. Medication costs account for a significant percentage of most health-system pharmacy budgets and continue to increase, with the latest data representing a 9.4% increase year over year.^1^ In addition to rising costs, suboptimal inventory management can lead to drug waste and medication errors, which can negatively impact patient outcomes.^2^ These challenges have led organizations to consolidate inventory and create integrated delivery systems to optimize expenses while preserving patient safety and the quality of patient care.^3-5^

Efficient medication inventory management is important to ensure the availability of medications, thus mitigating the risk of medication errors and adverse drug events. To optimize medication inventory management, it is essential to consider factors such as medication costs, drug shortages, recalls, manufacturing delays, and unexpected spikes in demand. Automation, real-time inventory tracking, and supply chain management can help address some of the common challenges and issues healthcare systems face when managing medication inventory.^6,7^ Medication inventory management solutions, such as inventory management software, automated central medication storage carousels, controlled substance vaults, and decentralized automated dispensing cabinets (ADCs), play a crucial role in maintaining patient safety, ensuring quality care, and optimizing expenses.^8^

One of the main benefits of inventory management systems is the ability to improve labor efficiency.^6-8^ With automated inventory management systems, healthcare organizations can reduce the amount of time and resources spent on manual inventory management.^6-9^ This automation allows healthcare workers to prioritize their critical work of caring for patients and allocate their time and resources more effectively, which leads to increased productivity and efficiency.^9^

Additionally, automated inventory management systems can help reduce waste by tracking inventory levels and expiration dates. With this information, healthcare workers can use medications before they expire and reduce the amount of excess inventory, thus leading to reduced waste and cost savings.^9,10^

These technologies require personnel to implement and maintain the medications stored within them.^6,7^ Historically, automated inventory management systems have relied on manual pharmacy interventions to improve clinical and operational outcomes. However, more recent trends have focused on novel approaches including artificial intelligence, data segmentation, and business intelligence dashboards.^11,12^ Further, in the 2022 ASHP Foundation Pharmacy Forecast report, 73% of pharmacists agreed that half of all health systems will have staff dedicated to pharmacy enterprise data analytics, and 71% of pharmacy leaders were willing to invest in digital health solutions to improve medication and inventory management by 2026.^13^

**Key Points**

- Connecting automated dispensing cabinets (ADCs), central automated pharmacy inventory systems, and automated controlled substance vaults with an inventory optimization analytics (IOA) tool may result in labor benefits and workflow improvements.
- Automating inventory management can yield ADC inventory storage cost reductions while also improving medication availability.
- Analytics solutions can be powerful operational tools but require adoption, understanding, and change management to optimize their impact.

Pharmacy leaders are challenged to meet these demands while being required to minimize drug spending and improve efficiency in their operations. Managing the pharmacy supply chain is complex, and automation tools that can simplify key processes in the system have yet to be ubiquitously deployed in healthcare enterprises. The purpose of this study was to assess a comprehensive inventory management solution’s ability to standardize inventory data and processes to reduce costs, improve labor efficiency, and ensure medication availability.

## Materials and methods

### Setting

The study was conducted in a 15-hospital integrated delivery network (IDN) in the eastern region of the United States. We included 11 acute care institutions in south and central North Carolina. The IDN offers a full range of clinical services and is staffed by approximately 35,000 employees, with around 750 reporting directly into the pharmacy department.

Inventory management is centralized and maintained at an IDN level. Procurement of medications and optimization of inventory levels are completed by a team consisting of 28 procurement and 7 automation specialists. Physical receiving and movement of medications is completed at the facility level and subject to facility variability in ADC device orientation and refill frequency. The centralized team oversees all standard formulary and periodic automated replenishment (PAR) level changes while providing governance over disparities in inventory management practices.

### Study design and methods

For the purposes of the study, the authors investigated the impact of implementing an enterprise inventory optimization (EIO) solution through various modules within an enterprise. The EIO solution consists of the following technologies: decentralized ADCs (BD Pyxis MedStation ES; Becton, Dickinson and Company [BD], San Diego, CA), automated central medication systems (BD Pyxis Logistics) utilized with or without storage carousels (Pharmacy Vertical Carousel; SencorpWhite, Hyannis, MA), controlled substances vaults (BD Pyxis CII Safe), and an inventory optimization analytics (IOA) software (BD HealthSight Inventory Optimization). The IOA software is an analytics solution intended to automate historically manual optimization processes including PAR changes, unloads, and destocks. The IOA solution consolidates visibility of inventory within the ADCs, carousels, and controlled substances vaults.

This article describes a mixed-method, postmarket, single-enterprise, multicenter, institutional review board–exempt, observational study designed to assess the impact of the EIO solution on labor, inventory management costs, and stockouts. Data collection took place between January and October 2022. The intervention was defined as implementation of the IOA software, as it was the last solution to be deployed and connected data from the other core solutions. Between the intervention and postintervention data collection, the investigators provided each site with a 90-day adoption period, allowing users the opportunity to acclimate to the new technology and workflows. As part of software implementation, pharmacy administrators defined targets within the system for minimum and maximum supplies on hand for ADCs; the administrators elected to follow the vendor recommendation of 1 day at minimum and 5 days at maximum for the duration of the study.

#### Study endpoints

**Figure 1.**
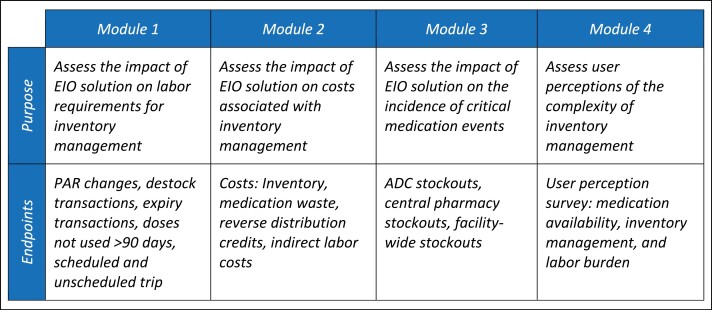
Overview of study modules with research goals and metrics.

The study was divided into 4 modules, each with an objective and associated endpoint. An overview of the modules and endpoints can be found in Figure 1. We combined transactional data from the EIO solutions and survey responses to complete the data set for each phase. Given variability in data requirements for the calculations built into the models, retrospective data was pulled for different time periods between the study modules.

The first module was designed to assess the impact of the technology on labor requirements for inventory management. The pharmacy captured time and human resource requirements for tasks before and after software implementation, such as PAR level adjustments and removal of expired medications. Labor was calculated utilizing a patent-pending algorithm (World Intellectual Property Organization application 2022/271384 A1) that identified ADC refill trips as scheduled (defined as a routine medication delivery at a specified time) or unscheduled (a delivery required because of an acute need for a medication). The same proprietary algorithm parameters were used for both the pre- and postintervention phases.

The second module was designed to assess the impact of the EIO solution on direct costs associated with inventory management, such as waste or expired medications. Standard ADC software functionality, such as outdate tracking, was enabled prior to preintervention data collection. Transactional data was obtained from the automated solutions and supplemented with cost data from the health system’s centralized purchasing department. Individual medication unit costs were obtained at the end of the study and applied to financial calculations in both the pre- and postintervention periods. During data analysis, there were a few items excluded when calculating inventory valuation. Those items included insulin and items with a maximum PAR value of 9,000 or more. Rationale for this exclusion can be found within the “Limitations” section of the Discussion.

The third module was to assess the impact on the incidence of critical medication events, such as stockouts. Transaction and inventory reports from the automated solutions were utilized to compile endpoints data.

Finally, the fourth module was to assess user perceptions of the complexity of inventory management based on responses provided on a 5-point Likert scale after software implementation. The perception survey was deployed to the centralized analytics and procurement teams as well as the pharmacy leaders at each site studied (a total of 43 surveys were distributed).

#### Statistical analyses

Descriptive statistics were calculated via Microsoft Excel (Microsoft Corporation, Redmond, WA) and reported to describe primary outcomes before and after the implementation. Student *t* tests were performed for all relevant statistical testing using Python version 3.6+ (Python Software Foundation, Beaverton, OR) and Microsoft Excel. Perception survey results were obtained and analyzed using Qualtrics (Silver Lake; Qualtrics, Seattle, WA), SAS statistical analysis software (SAS Institute, Cary, NC), and Microsoft Excel.

## Results

The study sites had various bed capacities ranging from 22 to 908 beds, with a total of 2,218 hospital beds (Table 1). Six hundred ADCs were included in the analysis, with an average of 1,363,605 monthly dispenses occurring in the preimplementation period and 1,300,341 transactions occurring in the postimplementation period (Table 1).

**Table 1.** Facility Characteristics and Inventory/Dispense Data by Study Period

Facility	Bed count	ADC count	Average adjusted patient days/month		Unique inventory items^a^		Average total dispenses monthly	
			Pre	Post	Pre	Post	Pre	Post
1	59	28	5,123	4,793	1,593	1,627	54,185	51,848
2	36	21	1,591	1,974	1,187	1,245	31,097	32,656
3	908	142	31,480	26,639	2,389	2,428	468,253	437,670
4	127	52	8,499	7,984	1,796	1,788	79,903	85,504
5	50	26	3,787	3,403	1,608	1,572	47,335	44,855
6	123	41	10,213	8,743	1,755	1,795	105,790	94,448
7	22	22	1,570	1,325	992	996	17,700	18,609
8	28	20	3,718	3,335	1,435	1,498	35,188	31,938
9	598	164	15,168	22,417	2,509	2,482	338,016	324,807
10	189	57	10,990	10,246	2,034	2,062	125,974	126,214
11	78	27	5,538	4,912	1,798	1,799	60,164	51,792
Total	2,218	600	97,677	95,771	19,096	19,292	1,363,605	1,300,341

Abbreviation: ADC, automated dispensing cabinet.

^a^Number of unique medication identifiers documented.

### Module 1 (labor)

**Figure 2.**
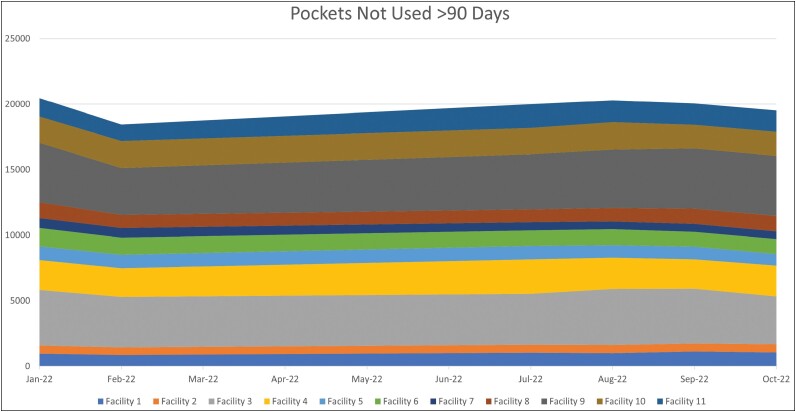
Ten-month trend in numbers of automated dispensing cabinet pockets not used for more than 90 days at the 11 study facilities.

As expected, supplying automated recommendations resulted in increased average daily PAR changes in 8 out of 11 facilities (73%), with 4 (36%) having a statistically significant increase (Table 2). We observed mixed results for the average daily expired and destock transactions, with 6 facilities (55%) showing decreases, 4 (36%) showing increases, and 1 (9%) having no change. When comparing the 11 facilities, there was no observable change in the overall trend of ADC pockets with no activity after 90 days (Figure 2).

**Table 2.** Inventory Management Transaction Trends by Study Period^a^

Facility	Average daily PAR level adjustments			Average daily expiry transactions			Average daily destock transactions		
	Pre	Post	*P* value	Pre	Post	*P* value	Pre	Post	*P* value
1	2	7	0.13	19	20	0.88	4	4	0.997
2	0	6	0.01	35	20	0.10	2	4	0.28
3	10	90	0.01	162	193	0.01	27	26	0.70
4	14	12	0.69	34	32	0.79	11	12	0.42
5	1	10	0.01	15	15	0.94	6	6	0.70
6	4	12	0.03	24	25	0.87	14	13	0.45
7	0	3	0.25	10	7	0.22	2	3	0.25
8	5	3	0.60	23	20	0.56	21	18	0.03
9	8	27	0.20	85	89	0.79	28	24	0.49
10	22	20	0.86	47	37	0.30	14	8	0.43
11	6	15	0.12	94	52	0.04	1	0	0.14
TOTAL	72	205		548	510		130	118	

Abbreviation: PAR, periodic automated replenishment.

^a^The preimplementation period was January 1 to February 24, 2022. The postimplementation period was September 11 to October 11, 2022.

When evaluating patient care area storage locations, a total of 7 out of the 11 sites (64%) showed a decrease in average daily unscheduled ADC refill trips, and 5 out of the 11 sites (45%) showed a decrease in average daily scheduled ADC refill trips (Table 3). Five out of 11 sites (45%) showed a statistically significant reduction in average total refill trips. At an enterprise level, we saw a reduction in average total refill trips of about 23 trips per day. Conservatively estimating an average of 15 minutes per refill trip, this reduction would provide a labor avoidance of 2,099 hours per year, or 1 enterprise full-time equivalent (FTE).

**Table 3.** Overview of ADC and Central Pharmacy Labor Metrics by Study Period

Facility	Average daily scheduled ADC refill trips		Average daily unscheduled ADC refill trips		Average total daily ADC refill trips			Average daily central pharmacy picks			Average daily central pharmacy batch picks		
	Pre	Post	Pre	Post	Pre	Post	*P* value	Pre	Post	*P* value	Pre	Post	*P* value
1	6	13	13	6	19	19	0.82	164	162	0.70	8	13	<0.001
2	5	5	6	6	11	11	0.58	116	122	0.12	30	35	0.07
3	49	47	45	39	94	86	<0.001	1,064	852	<0.001	888	956	<0.001
4	12	13	31	25	43	38	<0.001	279	290	0.11	24	36	<0.001
5	5	8	8	9	13	17	<0.001	156	156	0.94	43	28	<0.001
6	15	14	21	17	35	31	<0.001	360	332	<0.001	40	51	<0.001
7	4	4	2	2	7	7	0.66	61	58	0.53	29	29	0.83
8	8	7	23	26	31	34	0.11	106	103	0.40	9	7	0.004
9	38	32	63	60	101	92	0.004	928	931	0.87	252	339	<0.001
10	15	17	17	16	32	32	0.96	390	420	<0.001	98	147	<0.001
11	10	9	13	10	22	18	<0.001	233	218	0.003	32	34	0.53
Total	167	169	242	216	408	385		3,857	3,644		1,453	1,675	

Abbreviation: ADC, automated dispensing cabinet.

^a^The preimplementation period was December 27, 2021, to February 24, 2022. The postimplementation period was July 14 to October 11, 2022.

In the central pharmacy storage locations, 6 out of the 11 sites (54%) saw a reduction in unscheduled, nonbatch medication picks (eg, refilling a stockout outside of the scheduled fill), with 4 (36%) sites seeing a statistically significant reduction. Eight out of the 11 sites (73%) demonstrated an increase in the number of scheduled batch picks from the central pharmacy (Table 3).

### Module 2 (cost and inventory management)

Six out of 11 sites (55%) saw an increase in their total inventory costs within the automated storage location, with the overall enterprise inventory increasing $195,239 in the postintervention period. Increased inventory valuations were largely reflective of increases in the central pharmacy (64% of sites), whereas the biggest contributor to the inventory decreases was ADCs. Nine sites (82%) had a decrease equating to a reduction of over $680,000 (from $7,072,684 before implementation to $6,392,677 post implementation) among enterprise ADCs (Table 4). Eight sites (73%) saw an increase in the average daily cost of expired medications, while 8 (73%) saw a decrease in reverse distribution credits. There was an overall increase in average daily purchase order costs for all medications (ie, those stored in automated storage locations and those stored outside automation), indicating higher acquisition costs in the postintervention period.

**Table 4.** Pre-Post Comparison of Daily Inventory, Expired Medication Transactions, and Purchase Orders and Reverse Distribution Credits

	Average daily inventory on hand									Average daily cost of expired medications (all devices)		Average daily purchase orders (all medications),	Average daily reverse distributor credits (all medications),
	All devices			ADC devices			Vault and central inventory system						
Facility	Pre	Post	*P* value	Pre	Post	*P* value	Pre	Post	*P* value	Pre	Post	% increase ($) from pre to post	% decrease ($) from pre to post
1	$944,030.51	$828,722.34	<0.001	$499,252.05	$393,883.73	<0.001	$444,778.46	$434,838.61	0.28	$71.73	$213.54	64	98
2	$579,495.03	$531,763.57	<0.001	$130,411.12	$101,972.30	<0.001	$449,083.91	$429,791.27	0.15	$126.10	$115.66	140	100
3	$6,008,706.29	$6,405,269.24	<0.001	$1,656,733.95	$1,495,656.35	0.19	$4,351,972.34	$4,909,612.89	<0.001	$681.33	$935.31	51	85
4	$1,296,442.58	$1,456,109.69	<0.001	$793,078.12	$954,203.58	<0.001	$503,364.46	$501,906.10	0.75	$85.13	$547.54	31	70
5	$1,170,948.13	$1,191,172.77	0.14	$252,876.90	$208,838.91	<0.001	$918,071.23	$982,333.86	<0.001	$157.68	$163.87	47	93
6	$1,214,403.03	$1,265,432.27	<0.001	$675,363.82	$646,559.65	0.03	$539,039.21	$618,872.62	<0.001	$154.98	$1,035.12	128	39
7	$172,109.07	$183,629.37	<0.001	$51,096.04	$45,841.11	<0.001	$121,013.03	$137,788.26	<0.001	$115.04	$45.66	–7	–6
8	$1,213,071.79	$1,242,041.60	0.01	$580,390.80	$583,615.75	0.69	$632,680.98	$658,425.85	<0.001	$144.63	$180.06	72	53
9	$4,238,695.91	$4,173,865.28	0.89	$1,412,824.35	$1,249,064.82	<0.001	$2,825,871.56	$2,924,800.47	0.17	$2,056.17	$660.65	23	–441
10	$1,698,865.03	$1,502,305.21	<0.001	$647,850.26	$381,712.42	<0.001	$1,051,014.77	$1,120,592.79	<0.001	$184.21	$303.34	–22	NR
11	$816,224.16	$767,920.14	0.09	$372,806.98	$331,328.65	0.14	$443,417.19	$436,591.49	0.02	$133.21	$263.23	137	94
Total	$19,352,991.53	$19,548,231.48		$7,072,684.39	$6,392,677.27		$12,280,307.14	$13,155,554.21		$3,910.21	$4,463.98		

Abbreviations: ADC, automated dispensing cabinet; NR, not reported.

^a^The preimplementation period was January 25 to February 24, 2022. The postimplementation period was July 14 to October 11, 2022.

### Module 3 (medication availability)

Out of the 11 sites, 7 (64%) had a decrease in the patient care area or ADC stockout percentage and all 11 sites had a reduction in the central pharmacy carousel stockout rate (Table 5). Eight of the 11 sites (73%) saw a reduction in the central controlled substances vault stockout, and 8 sites (73%) showed a reduction in the overall facility-wide stockout percentage (ie, the proportion of stockouts that resulted in complete facility supply exhaustion).

**Table 5.** Medication Stockout Rates for ADCs, Central Pharmacy (Inventory System With Carousel and Controlled Substance Vault Technology) and Facility-Wide Stockout Rate

Facility	Patient care area (ADC) stockout %			Central pharmacy (carousel) stockout %			Central pharmacy (CS vault) stockout %			Facility-wide stockout %	
	Pre	Post	*P* value	Pre	Post	*P* value	Pre	Post	*P* value	Pre	Post
1	0.73	0.69	0.47	7.25	6.39	0.13	2.65	2.85	0.82	0.14	0.13
2	1.18	1.26	0.6	4.41	3.52	0.55	2.12	0.46	0.08	0.14	0.12
3	0.95	0.78	<0.001	0.84	0.55	<0.001	4.7	1.8	<0.001	0.08	0.04
4	0.53	0.58	0.53	5.48	3.09	0.03	3.71	2.59	0.27	0.25	0.20
5	1.02	0.74	<0.001	4.18	4.01	<0.001	5.52	4.43	0.39	0.17	0.13
6	0.6	0.48	0.02	3.4	1.61	<0.001	10.36	2	<0.001	0.20	0.06
7	1.39	1.04	0.08	5.29	3.79	0.01	0	0.68	0.11	0.17	0.21
8	0.77	0.76	0.96	6.37	4.23	0.03	1.27	1.1	0.85	0.37	0.18
9^b^	0.68	0.68	0.99	2.45^b^	1.63^b^	0.05	0.71	0.56	0.43	0.07	0.06
10	0.51	0.62	0.01	2.49	1.33	0.48	2.08	1	0.04	0.07	0.07
11	0.89	0.54	<0.001	3.36	2.32	<0.001	0.79	1.68	0.1	0.11	0.13

Abbreviations: ADC, automated dispensing cabinet; CS, controlled substance.

^a^The preimplementation period was January 25 to February 24, 2022. The postimplementation period was September 11 to October 11, 2022.

^b^Utilization of a physical carousel.

### Module 4 (user perception)

**Figure 3.**
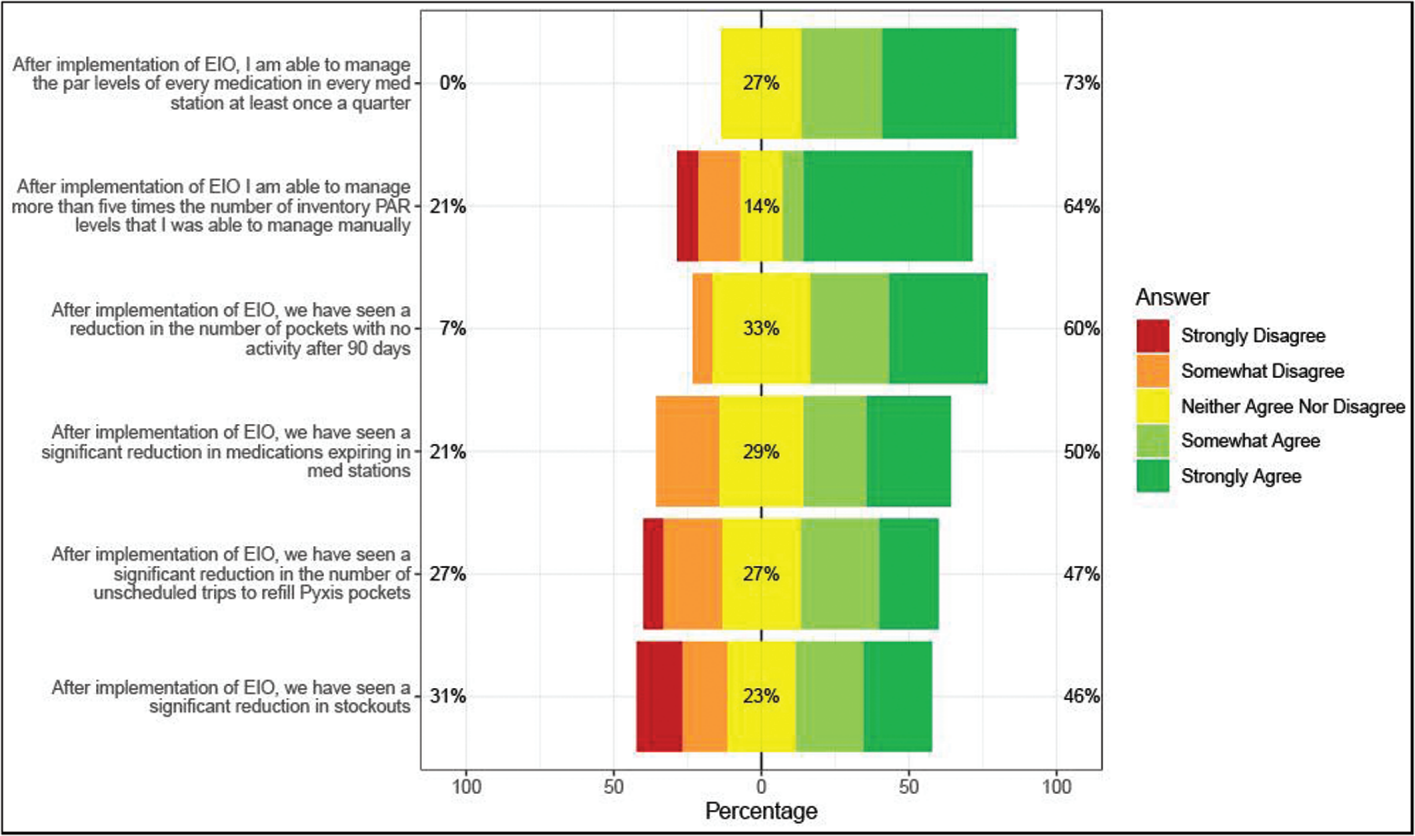
Results of user perception survey. EIO indicates enterprise inventory optimization; PAR, periodic automated replenishment.

Overall, the results of the user perception survey complimented the objective metrics (Figure 3). Seventy-three percent of all users (n = 11/15) indicated they could manage PARs once a quarter, with 64% saying they could manage 5 times more than before the intervention (the actual enterprise increase was 2.84 times the baseline number). Forty-seven percent of respondents indicated a reduction in unscheduled refill trips, and 60% felt there was a reduction in pockets not used for more than 90 days. When asked about pharmacy waste, 50% of users felt there was a decrease in expired medications after solution implementation. Finally, 46% of respondents (n = 7/15) agreed that after solution implementation, they saw a significant reduction in stockouts.

## Discussion

The study results indicate that with connected EIO solution utilization, organizations can standardize how inventory data is viewed and managed to improve labor efficiency, reduce costs, and ensure medication availability. We observed two major findings in our assessment of the labor impact of the EIO solution: Many sites saw a reduction in overall ADC refill trips and a shift towards increased scheduled batch picks. The hours avoided by optimizing refill trips could be interpreted as equating to a savings of either $37,924 (based on the geographic median hourly pharmacy technician wage of $18.07)^14^ or more than 1 enterprise FTE that could be reallocated to more value-added activities. We also investigated whether this decrease in refill trips would impact the central pharmacy dispensing workload. Our central pharmacy results show an overall reduction in nonbatch dispenses and an increase in batch dispenses. This could indicate a postintervention shift from a reactionary workflow, wherein critically low and stocked-out inventory values were driving workload, to a more predictable and scheduled picking cadence. Any labor support that technology may offer is especially important during current labor force challenges, with a recent survey showing that 91.8% of hospitals have a shortage of experienced pharmacy technicians.^15^

From a cost perspective, the $680,000 reduction in ADC inventory value is significant given the short study period. While the overall inventory value increased by $195,000, we suspect this may have been indicative of excess ADC inventory transported back to the central pharmacy. This assumption is supported by the increased central pharmacy valuation during the postimplementation period. We believe this to be a temporary increase that will deplete over time as medication inventory is consumed. The inventory changes were even more substantial, as there was a health-system policy change to maintain a 5-day supply of medications on hand in ADCs post intervention versus 3 days on hand during the preimplementation data collection period. While mixed results were seen regarding waste reduction, a decrease in expired medication transactions and reverse distribution reimbursements could indicate optimized deployment of inventory. Additionally, the large increase in enterprise purchase order costs combined with the cost reduction of automated solution medication storage suggests external factors, such as high-priced infusions and seasonality, may have contributed to conflicting results. While confounding factors prevent the authors from affirming total pharmacy savings, we note the possibility that inventory management by automation may result in financial benefits.

Our final group of endpoints surrounded medication availability. Reductions in inventory must be weighed and balanced with medication availability for patient care. In this study, most of the sites saw a stockout reduction within the ADCs, including 4 statistically significant reductions. These results are meaningful given that ADC medication availability has previously been linked to not only medication safety and pharmacy inventory management efficiency but also nursing satisfaction and labor benefits.^16^ It is important to note facilities had employed policies to optimize ADC stockout percentage to an industry standard of less than 1% prior to the intervention, so statistically and operationally significant reductions were not necessarily expected.^6^

The biggest change observed was the reduction of stockout percentage within centralized pharmacy automation at all facilities. The authors are unaware of an industry benchmark for this metric, but our small sample size suggests a target of less than 3% appears achievable by facilities. Finally, a new metric was introduced: facility-wide automation stockout percentage. We again saw a directional improvement in facility-wide automation stockout percentage at an enterprise level. While the total reduction percentages were very small, we feel the stockouts are more clinically relevant as they represent a potential delay in therapy or forced substitution with each occurrence. This metric could become a standard target, and we encourage technology vendors to incorporate it into standard reporting and analytics.

Finally, the study sought to understand user acceptance of this integrated EIO solution. Understanding that user perception surveys are inherently subjective, our goal was to align survey data with objective metrics as closely as possible. For most survey responses, the objective improvements matched the overall benefits perceived by pharmacy users. The biggest incongruent response was surrounding medication pockets not used within 90 days; 60% of users felt there was an improvement, while the data showed no signs to support that perception. While the exact reason for this discrepancy is unclear, the low sample size or wording of the question may have influenced the final perception result.

### Limitations

The limitations of this research highlight the complexity of implementing and optimizing a new inventory management system in a healthcare setting. Despite the recognized advantages of leveraging modern analytics and algorithms, several limitations were identified. One of the main constraints was the level of understanding and experience required to maximize system usage. Shifting from snapshot reporting to a global overview or advanced visualization with new technology requires transitional time for change adaptation, an adjustment period for pharmacy team members to acclimate to the new system. Further data collection would be required to determine if 90 days was a suitable technology adoption and change management period.

Second, the study was constrained by the lack of readily available and consistent data among the disparate technologies. Lack of real-time reporting hindered the ability to understand and demonstrate the potential optimizations that can be achieved using the software. For example, cost information within the automated system was provided as a snapshot rather than up-to-date wholesaler information for each National Drug Code. This is another possible explanation for cost discrepancies between the pre- and postimplementation periods, as inflationary and shortage trends were largely ignored during the study period. While this is an industry-wide technology limitation, we recommend that investment in analytics solutions should encompass robust reporting capabilities to ensure meaningful outputs that can inform strategic decision-making. Additionally, data for pockets not used within 90 days was not conducive to analysis as a date range but instead relied on calendar month reporting, which made it difficult to visualize any significant change during the defined study period.

Third, there were two additional modules (ie, modules 5 and 6) included in the original study protocol. However, the study did not include some technology solution analytics capabilities, such as the shortage management and formulary mapping services. As this base functionality was essential to evaluating endpoints in study modules 3 and 4, data could not be collected and therefore the study modules were omitted from the final study.

Fourth, as mentioned in the discussion of methodology, there were certain items that were excluded from cost calculations. As insulin was typically dispensed per unit or milliliter when stored in an ADC but by vial when stored in a carousel, there was not a clean way to assume cost when analyzing data. Additionally, automated storage systems may contain inventory counts to represent general stock items such as fluid bags or keys. For this data set, those items were identified as having a maximum PAR value greater than or equal to 9,000 units. While these items have a cost and would have contributed to inventory valuation, their exclusions had a negligible impact on overall statistical significance.

Lastly, the study timeframe may not have been sufficient to truly reflect cost and waste avoidance indicators. Longer lead and lag times could offer a more comprehensive understanding of the true impact of the system. This is especially true given the health-system ADC policy change from 3 to 5 days on hand. In addition, patient care seasonal variations might also have been a factor in the differences in inventory during the two study periods. However, the authors believe this is representative of real-life scenarios and the data presented is still relevant.

Despite the limitations, the study underscores the value of a comprehensive, multi-faceted approach to inventory management in healthcare. The results demonstrate that deploying comprehensive enterprise technology solutions can improve management of pharmacy inventories and reduce waste of expiring or unused drugs. The operational benefits underscore the solution’s value in real-world applications. It enables a clear visualization of the impact of the new technology, enhancing our understanding of its utility in a healthcare setting.

Moreover, the research validates the importance of conducting systematic analysis on every new technology implemented in a healthcare setting to ensure its suitability for the task at hand. The potential benefits of the software, particularly its usability, can only be harnessed through comprehensive training and education to ensure appropriate use. This study contributes to a growing body of evidence emphasizing the importance of a data-driven, technologically informed approach to inventory management in healthcare.

## Conclusion

Although medication inventory management is often believed to be a balance between labor, cost, and availability, this study proposes that all three may be simultaneously improved with the use of connected automation and analytics. Further studies are encouraged to expand on this research, particularly in exploring longer lead and lag times to truly understand the full extent of the impact and effectiveness of inventory management systems.

## Previous affiliations

At the time of the work performed, Amanda Prochazka was affiliated with Corporate Pharmacy, Novant Health.
